# How to Promote Low-Carbon Economic Development? A Comprehensive Assessment of Carbon Tax Policy in China

**DOI:** 10.3390/ijerph182010699

**Published:** 2021-10-12

**Authors:** Weijiang Liu, Yangyang Li, Tingting Liu, Min Liu, Hai Wei

**Affiliations:** 1 Center for Quantitative Economics, Jilin University, Changchun 130012, China; liuwj@jlu.edu.cn; 2 Northeast Revitalization and Development Research Institute, Jilin University, Changchun 130012, China; 3 Business School, Jilin University, Changchun 130012, China; 18326921663@163.com (T.L.); minliu19@mails.jlu.edu.cn (M.L.); 4 School of Cyber Security, Gansu University of Political Science and Law, Lanzhou 730070, China; weihai94@163.com

**Keywords:** carbon tax, low-carbon economy, CO_2_ emissions, double dividend, CGE model, tax neutrality, carbon tax recycling system

## Abstract

Facing the increasingly severe environmental problems, the development of a green and sustainable low-carbon economy has become an international trend. In China, the core issue of low-carbon economic development is effectively resolving the contradiction between the exploitation and utilization of fossil energy and greenhouse gas emissions (mainly carbon emissions). Based on the SAM matrix, we established a static Computable General Equilibrium (CGE) model to simulate the impact of carbon tax policies on energy consumption, carbon emissions, and macroeconomics variables under 10, 20, and 30% emission reductions. Meanwhile, we analyze the impact of different carbon tax recycling mechanisms under the principle of tax neutrality. We find that the carbon tax effectively reduces carbon emissions, but it will negatively impact economic development and social welfare. A reasonable carbon tax recycling system based on the principle of tax neutrality can reduce the negative impact of carbon tax implementation. Among the four simulated scenarios of carbon tax cycle, the scenario of reducing residents’ personal income tax is most conducive to realizing the “double dividend” of carbon tax.

## 1. Introduction

The fundamental way for humanity to cope with climate change and realize the coordinated development of energy, environment, and economy is to implement and develop a low-carbon economy [1,2]. The global economic growth and the gradual increase in production activities have accelerated carbon emissions [3]. At the same time, the rapid growth of carbon emissions has seriously threatened the survival of human beings and the sustainable development of the social environment [4]. In recent years, more than 30 countries around the world have established the goal of achieving carbon neutrality around the middle of this century [5]. As the world’s largest carbon dioxide emitter, China also pledged at the 2020 United Nations General Assembly to achieve carbon neutrality by 2060, and carbon dioxide emissions peaking before 2030. However, judging from the current situation, for countries in the process of high-speed industrialization, energy demand will show an upward trend for a long time. Therefore, controlling greenhouse gas emissions is still a key consideration in formulating environmental policies [6].

In order to promote the development of a low-carbon economy and reduce the impact of carbon emissions on the social environment, governments of various countries have begun to explore ways to reduce carbon emissions [7]. For example, implementing carbon taxes and carbon emission trading schemes are effective policy tools to reduce carbon emissions [8,9]. Among them, carbon tax policy has shown promising results in controlling carbon emissions [10,11,12]. The carbon tax is an environmental protection tax, which aims to reduce carbon emissions and alleviate global warming. Because the levy of a carbon tax will increase energy products’ prices, the demand for energy products will decrease, reducing carbon emissions [13]. Secondly, the market mechanism will encourage clean energy to achieve sustainable energy development and the double emission reduction effect of the carbon tax [14]. However, the carbon tax will also bring some negative effects [15], such as increasing the tax burden of enterprises and residents, affecting their social welfare. In order to reduce these adverse effects and ensure the carbon tax system’s implementation, reasonably using the incentive mechanism to design the carbon tax and integrating it into the existing tax system or adapting it to tax system reform is significant to improving the environment and strengthening the tax system’s income redistribution [16,17]. Therefore, when formulating carbon tax policies, it is necessary to determine a reasonable carbon tax recovery mechanism based on the principle of tax neutrality.

The principle of tax neutrality refers to keeping taxes and expenditures unchanged, achieving budget neutrality, minimizing distortions to the market as much as possible, and ensuring the market’s pure competitiveness. It does not aim to increase fiscal revenue, but returns most of the carbon tax revenue to taxpayers in the form of subsidies and compensation, thereby increasing the acceptability of the carbon tax system and reducing the impact of the carbon tax on social welfare. A carbon tax system that complies with the principle of tax neutrality will also reduce the “extra burden” of the carbon tax itself and the negative impact of the regressive nature of the carbon tax on taxpayers, especially low-income households. In the initial stage of the implementation of the carbon tax, to enhance the acceptability of carbon tax, the tax rate will usually be gradually increased from a lower level, so as to give play to the emission reduction function of the carbon tax system, which is in line with the principle of tax neutrality and the goal of sustainable economic development. The “tax neutrality principle” requires that taxpayers’ tax burdens be balanced and stable at the macro level. Besides, China is implementing “structural tax cuts”. Therefore, taxpayers’ tax burdens in other areas should be reduced while carbon taxes are levied; it will realize the “double dividend” of improving enterprises’ and residents’ welfare and enhancing carbon emission reduction. The carbon tax recycling mechanism ensures that the overall income and expenditure remain unchanged and conform to tax neutrality. Meanwhile, it can reduce the impact of other distorting taxes and achieve tax burden shifting [18]. It also considers the environmental and economic goals of carbon tax collection, as making it more systematic can achieve the double dividend of reducing emissions and increasing social welfare. A carbon tax will only be accepted and supported by the public if it can achieve the goal of reducing carbon emissions. The double dividend effect of the carbon tax recycling mechanism further guarantees public support, making the implementation of the carbon tax more likely.

As a favorable tool for policy analysis, scholars have adopted different CGE models (such as static CGE, dynamic CGE, etc.) to study the effect of carbon tax policy implementation, confirming that the CGE model has significant advantages over other measurement tools in the field of policy simulation [19,20]. This paper conducts empirical analysis by establishing an environmentally Computable General Equilibrium (CGE) model for analyzing carbon tax policies. However, most previous studies have only analyzed the effects of the carbon tax and paid less attention to the synergy of multiple policies. In addition, these studies lack effective incentive policies to implement the carbon tax policy better. Additionally, they have seldom paid attention to the distortion of resource allocation caused by the additional burden of taxation. Differently, the contribution of this article is to uphold the principle of tax neutrality to design-related carbon tax simulations. In formulating the carbon tax revenue use mechanism, a carbon tax recycling system that reflects the principle of tax neutrality has been incorporated, and the revenue from the carbon tax will be used to reduce the tax burden of distorting taxes such as corporate income tax, so as to keep the overall tax burden from increasing. We contribute to incorporate the carbon tax, comprehensively analyzing carbon taxes’ socio-economic impact on the country’s low-carbon economy; meanwhile, we verify the existence of a double dividend. Finally, this article provides relevant policy recommendations for the government to design a reasonable carbon tax.

The study is structured as follows: Section 2 addresses the literature review. Section 3 outlines the methodology and data description. Section 4 presents the scenarios setting. The empirical results are presented in Section 5. Finally, Section 6 provides the discussion and conclusion.

## 2. Literature Review

### 2.1. Research on Influencing Factors of Carbon Emissions

Since the 21st century, China’s economy has developed rapidly, and the energy provided by fossil fuels has greatly promoted the development of industries. With the massive exploitation and use of fossil energy, global air pollution, and the greenhouse effect have affected human survival and sustainable development [21,22]. In order to control carbon emissions effectively, it is essential to conduct in-depth research on the factors affecting its emissions, and it has become a hotspot in energy and environmental policy research.

The signing of the “Kyoto Protocol” in 1997 brought China’s climate change research and governance into a new chapter [23,24,25]. Scholars around the world have conducted intense discussions on greenhouse gas emissions and governance. Among them, many scholars have demonstrated that the greenhouse effect has varying degrees of negative impact on different industries [26,27,28]. Pearce et al. [29] studied the impact of climate change on Employment in Australia and drew the conclusion that climate change may bring about employment growth and decline in different fields. Awan et al. [30] have suggested that firm environmental management capabilities at the production level have considerable value for addressing CO_2_ emissions and minimizing environmental pollution. It requires a varied degree of competencies, in the product life cycle stage at the level of process and product to ensure and improve pollution performance. Research on climate governance relies on the decomposition and calculation of reliable carbon emissions influencing factors. Zhao et al. [31] studied the relationship between China’s import and export and carbon emissions, confirming that the changing trend of low-carbon industry structure can effectively reduce carbon emissions growth.

In studying the influencing factors of carbon emissions, the decomposition identity of carbon emission factors proposed by Japanese scholar Kaya is widely used [32]. On this basis, Ang and Liu [33] proposed a new decomposition method—LMDII, which quantitatively decomposed the influencing factors of carbon emissions and confirmed that this method has a perfect effect on greenhouse impact emissions from various fields. For example, Liaskas et al. [34] use this method to analyze the influencing factors of industrial carbon dioxide emissions in E.U. countries. Zhang et al. [35] conduct a complete factor decomposition on China’s energy carbon dioxide emissions and emission intensity during 1991–2006. In terms of application, scholars have done a lot of research. For example, Schipper et al. [36] applied the AWD (Adaptive-Weighting-Divisia) method to analyze the carbon dioxide emission trends of 13 countries. It is believed that energy intensity and energy consumption structure can explain most carbon emission intensity changes for most countries. Davis et al. [37] used the AWD method to analyze the reasons for the decline in energy intensity and carbon emissions in the United States from 1996 to 2000 and believe that energy structure adjustment is not the main reason, but weather changes are the main reason.

Based on the above analysis, it can be concluded that energy consumption is closely related to carbon dioxide emissions. Implementing measures to control energy consumption, such as a carbon tax, is the most effective means to reduce carbon dioxide emissions. Next, we will review the current major carbon emission mechanisms and choose the emission reduction mechanism that best suits China’s national conditions.

### 2.2. Research on Carbon Emission Mechanism

Carbon taxes and carbon trading systems are carbon pricing policy tools commonly used by countries to promote carbon emission reduction, which will affect the cost of enterprises and individuals [38]. Currently, most countries have different emission reduction targets. The main reason is that countries are in different development stages, so the carbon emissions are also very different. As the world’s largest carbon dioxide emitter, some regions in China have begun to implement the carbon trading system. In recent years, as countries have become increasingly aware of carbon taxes, it is necessary to assess whether carbon taxes effectively achieve emission reduction targets [39]. Next, we mainly conduct a comparative analysis of the two main carbon emission reduction systems, carbon tax and emission trading.

From the perspective of implementation costs, the carbon tax implementation cost and supervision cost are lower than the carbon trading system [40]. The carbon tax can be directly implemented as a tax item of environmental protection tax, reducing the cost of use [41]. The carbon trading system lacks coercive force in carbon emission reduction. In the carbon trading environment, the cost of carbon emission reduction depends on the uncontrollable market, which easily brings unreasonable emission reduction costs and greatly reduces the emission effect [42]. From the perspective of economic impact, under the principle of tax neutrality, the carbon tax has the characteristics of “double dividend” [43,44]. The collection of the carbon tax is not only conducive to achieving carbon emission reduction and improving the environment, but also guiding companies to make business decisions that are conducive to their own development and environmental protection based on the determined carbon tax price, making the economy more efficient [45]. However, the carbon emission price that relies on the market price mechanism is uncertain and cannot provide companies with an accurate assessment basis. For social welfare, Timilsina and Shrestha [46] used a static CGE model to find that the carbon tax has a smaller impact on the total social welfare compared with other emission reduction policy tools. When carbon tax revenue is used to reduce the existing indirect tax rate of non-energy products, the impact on the total social welfare is minimal.

### 2.3. Research on CGE Model of Carbon Tax

The carbon tax has become an emission reduction measure strongly recommended by economists and international organizations to reduce greenhouse emissions. Beginning in the 1990s, many scholars started to use the CGE model to simulate national carbon taxes [19,20], and then analyze the impact of carbon taxes on the energy system and economic variables such as GDP, welfare, investment, imports, and exports. At present, the CGE model has become one of the most mainstream tools for analyzing energy, environmental, and climate policies globally.

Garbaccio et al. [47] used a dynamic CGE model to simulate and analyze the impact of the carbon tax on China’s economic development under the co-existence of a planned economy and a market economy. Shrestha and Marpaung [48] used the CGE model to study the impact of carbon taxes on the Indonesian power sector and proposed that the levy of carbon taxes can lead to a substantial increase in prices in the power sector and a significant decline in energy consumption. Wissema and Dellink [49] used the CGE model to analyze the impact of levying a carbon tax in Ireland on its economy and proposed that a carbon tax levying would significantly affect Irish consumption patterns. Allan et al. [50] used an energy-economy-environment CGE model to study Scottish carbon taxes’ economic and environmental impacts. The study found that carbon taxes may achieve double dividends when carbon tax revenues are recycled through income taxes. Lin and Jia [51] analyzed the impact of carbon tax policies on the energy environment and economy by constructing CGE models of different carbon tax usage scenarios. The study found that the negative impact of the carbon tax on GDP is acceptable, and the higher the carbon tax rate, the greater the carbon dioxide emission reduction of the carbon tax policy. Fu et al. [52] developed a CGE model to examine the social impact of carbon tax policy, finding that a carbon tax is a powerful driving force to reduce carbon emissions and promote the energy revolution.

In summary, domestic and foreign scholars have adopted the CGE model to conduct related research on the emission reduction effects of carbon tax policies, proving that the CGE model has significant advantages over other measurement tools in policy simulation.

This paper conducts empirical analysis by establishing an environmentally Computable General Equilibrium (CGE) model for analyzing carbon tax policies. Specific simulation research on the impact of carbon tax policies implemented to control carbon dioxide emissions on energy consumption, carbon dioxide emissions, and sectoral economic variables; analyze different carbon tax cycle policies’ impacts on China’s macroeconomic variables under the principle of tax neutrality, and explore how to realize double dividend. The above analysis can provide references for the implementation and policy formulation of China’s carbon tax policy.

## 3. Methodology and Data

### 3.1. Description of the CGE Model

**Figure 1:**
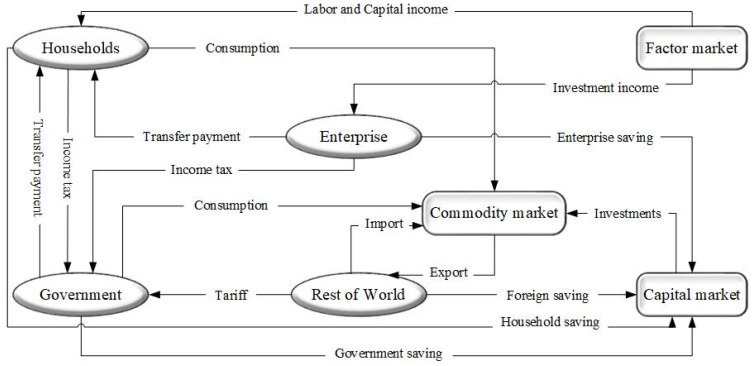
Skeleton of proposed CGE model.

The CGE model adopted in this study has been widely used to simulate and analyze energy and environmental policies [53,54,55], systematically analyzing policy’s impact under the general equilibrium framework. This model is based on Walras’ general equilibrium theory, with producers, consumers, government, and foreign sectors as the basic economic units, and it can analyze the interaction of one or more variable disturbances on other variables and the entire economy [56,57,58]. Figure 1 portrays the detailed model skeleton.

The main structure of the production module is a five-layer nested structure. Given the importance of energy inputs to carbon emissions and the substitution effect among different energy sources, the energy inputs (coal, oil, and gas) are described by Constant Elasticity of Substitution (CES) functions and form an energy synthesis factor. The energy synthesis factor is in turn combined with capital through the CES function to form the energy capital factor, like many other studies [59,60]. Then, combined with labor through CES function to form the energy-capital-labor factor, that is, the value-added composites. This allows substitution between various input factors. The energy-capital-labor factor and non-energy intermediate inputs are combined through a Leontief function to form sectoral outputs. Most research on the CGE model separates oil and natural gas, but the separation method is quite rough and does not consider the difference in intermediate inputs. The main fossil energy is coal, so this article does not separate the two industries.

Referring to previous studies [61,62], the trade module functions are the constant elasticity transformation (CET) and Armington functions, assuming the distribution of domestically produced products and the domestic demand. The household consumption function generally adopts the Stone-Geary utility function, but its parameters are difficult to estimate. At the same time, this article is a static analysis. Therefore, the household consumption function adopts a simple linear function form. The closed rule adopts the neoclassical closure rule, assuming that labor and capital prices are endogenous. The labor and capital market achieve full employment. The social welfare function is measured by Hichsian equivalent variation. Specifically, it is based on the commodity price before implementing the policy and measures the household utility level change after implementing the policy.

Carbon dioxide emissions associated with fuel combustion and carbon taxes are introduced into the environmental module. The carbon tax is levied on carbon dioxide emissions related to fuel. By calculating the total carbon tax ratio on each fossil energy to the total fossil energy demand, the Ad valorem tax rate for fossil energy is obtained.

### 3.2. Data Sources

#### 3.2.1. Source of Basic Data

The basic data of this CGE model is obtained from the 2017 social accounting matrix (SAM) table, which is based on China’s 2017 input-output table and relevant data on customs, tax revenues, international payments, and capital flows. The SAM table includes nine sectors^1^, three institutions (households, enterprises and government), and three production inputs (labor, capital and energy). The capital, government, and foreign inputs come from the 2018 China Statistical Yearbook (http://www.stats.gov.cn/tjsj/ndsj/, accessed on 6 October 2021) and the 2018 China Financial Yearbook (https://www.epsnet.com.cn/, accessed on 6 October 2021). Households savings are taken from the 2017 Flow of Funds Statement (http://www.stats.gov.cn/tjsj/ndsj/, accessed on 6 October 2021). The carbon dioxide emissions data come from the International Energy Statistics (https://www.eia.gov/, accessed on 6 October 2021) on the carbon dioxide emissions of China’s three fossil energy sources.

#### 3.2.2. Parameter Calibration

The model’s parameters that need to be calibrated mainly include substitution elasticity coefficient, share parameter, and carbon dioxide emission coefficient. The substitution elasticity coefficients in the production and trade function are generally obtained through econometric methods or consulting relevant experts. The setting of alternative elastic parameters in this article mainly refers to previous literature [63]. The shared parameter is calibrated by the elasticity of substitution and the base year data of the variable. Using statistical data from the International Energy Statistics, the carbon dioxide emission coefficient is calculated based on the fossil energy sources’ carbon dioxide emissions and the actual energy consumption.

## 4. Scenarios Setting

### 4.1. Carbon Tax Design

In the climate policy simulation of this model, carbon dioxide emissions can be directly calculated. That is why we use carbon dioxide emissions as the basis for calculating the carbon tax. The final investment and consumption demand of fossil energy accounts for a relatively small proportion of the total demand for fossil energy. Therefore, this article assumes that no carbon tax is levied on the final demand part, and only the intermediate input of fossil energy is taxed. The specific carbon tax design is as follows:

(1) $$CTAX_{i}=tc\cdot \sum_{j}E_{i,j}\cdot \theta_{i}$$

(2) $$CTAX_{j}=tc\cdot \sum_{i}E_{i,j}\cdot \theta_{i}$$

(3) $$TCTAX=\sum_{i}CTAX_{i}$$

where, $CTAX_{i}$, $CTAX_{j}$, and TCTAX are the carbon tax levied on the intermediate input of fossil energy i, carbon tax levied by sector j and the total carbon tax, respectively; tc represents the amount of carbon tax levied per ton of carbon dioxide emissions, that is, carbon tax; $E_{i,j}$ represents the energy input of fossil energy i in the sector j; $\theta_{i}$ is the unit energy carbon dioxide emission coefficient of energy i.

Based on the above calculations, we can convert the carbon tax rate of fossil energy into an ad valorem tax rate, that is, the carbon tax ratio on certain fossil energy to the value of the domestic demand for that fossil energy. The calculation formula in Equation (4).

(4) $$tc_{i}=\frac{CTAX_{i}}{PQ_{i}\cdot QQ_{i}}$$

where, $PQ_{i}$ and $QQ_{i}$, respectively, represent the demand and price of fossil energy i. As a result, the price of fossil energy demand will become $\left(1+tc_{i}\right)\cdot PQ_{i}$. This will increase the cost of using fossil energy in the production function energy input, and government revenue will increase due to carbon tax.

### 4.2. Simulation Scenario Design

#### 4.2.1. Carbon Emission and Energy Simulation Analysis

The ad valorem tax rates levied on different fossil energy sources are affected by the energy’s carbon dioxide emission coefficient. The price of fossil energy and the production cost of enterprises will increase due to the levy of carbon taxes, affecting the consumption demand of different energy sources. Meanwhile, the ratio of fossil energy input to total input in different sectors is different. The production functions and the substitution elasticities of various production factors are inconsistent, affecting the fossil energy demand of different sectors. Therefore, we simulated and analyzed the impact of the carbon tax on various energy, sector, and macroeconomic variables under the emission reduction scenarios of 10, 20, and 30% reduction in total carbon dioxide emissions, respectively.

#### 4.2.2. Carbon Tax Recycling Simulation Analysis

Levying a carbon tax can improve the environment, but it will have an adverse impact on economic development, residents’ income, and social welfare. At the same time, it will increase corporate production costs and decrease corporate profits. For producers, it will increase investment in alternative energy sources, which will improve the efficiency of fossil energy utilization and the development of new energy. For consumers, rising energy prices will cause them to look for alternatives such as clean energy. If there is no good spending policy when the carbon tax is levied, its redistributive effect will be limited even if the policy is already very sound. The makers of carbon taxes should shift the tax burden to those directly responsible, so as to provide effective incentives to reduce carbon emissions.

The principle of tax neutrality requires that taxpayers’ tax burdens are balanced and stable at the macro level. The carbon tax recycling mechanism ensures that the overall income and expenditure remain unchanged, in line with the principle of tax neutrality. Using carbon dioxide taxes to reduce the tax rates of existing taxes (such as income taxes, capital taxes, etc.) can reduce the impact of carbon taxes on related industries. At the same time, we will realize the double dividend of environmental taxes [26]. The first dividend is to improve the efficiency of fossil energy utilization, thereby reducing environmental pollution and improving the ecological environment. The second dividend reduces other tax rates or government transfer payments while imposing a carbon tax, increasing social welfare, and enhancing corporate competitiveness.

In summary, this study starts from the principle of tax neutrality, assuming three carbon tax recycling scenarios to compare the impacts on the social economy and verify the carbon tax double dividend. In the simulation analysis, Business as Usual (BaU) leverages a carbon tax under the scenario of reducing carbon dioxide emissions by 20%. The simulation scenario is to set up different carbon tax recycling methods. The specific settings for the simulating scenarios are designed, as listed in Table 1.

## 5. Results

### 5.1. Energy and Carbon Emissions Simulation Results

#### 5.1.1. Energy Effect

With the increase of carbon dioxide emission reduction, the carbon tax level gradually increases, as shown in Table 2. Meanwhile, the ad valorem carbon tax rate levied on fossil energy (coal, oil, and natural gas) gradually increases, with coal having the highest tax rate. When the emission reduction reaches 30%, coal’s ad valorem tax rate will reach 41.95%. As for oil and natural gas, it is relatively low. In different emission reduction scenarios, fossil energy contributes differently to the total CO_2_ emission reduction. We can see that the reduction task is mainly coal energy. This finding is consistent with other studies [51,52].

When carbon dioxide emission reductions decrease, the reduction ratio of energy consumption also increases. Among them, the reduction ratio of coal is the largest. When the emission reduction falls from 30 to 10%, the reduction ratio of coal consumption drops from 36.14 to 12.22%. However, the degree of electricity decline is not large, with a change of only 1.02%.

#### 5.1.2. Sectoral Effects

**Figure 2:**
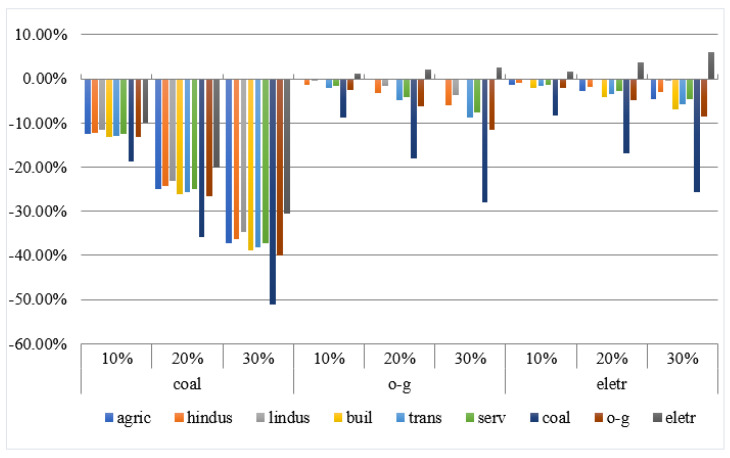
Consumption of each sector. The nine sectors and their classification are as follows: Agriculture (agric), Heavy industry (hindus), Light industry (lindus), Building industry (buil), Transportation industry (trans), Service industry (serv), Coal industry (coal), Oil and gas industry (o-g), Electricity industry (eletr).

Under different emission reduction scenarios, various sectors’ demand for fossil energy (coal, oil, and natural gas) and electricity is depicted in Figure 2. The bars express the percentage change in sectors’ energy consumption. The demand for coal in all sectors has fallen sharply. With the increase in emissions reductions, the extent of the decline has continued to increase. Taking the transportation industry as an example, when the carbon dioxide emission reduction is 10%, the demand for coal decreased by 12.86%; when the emission reduction is 30%, the sector’s coal consumption reduces by 38.16%. The oil and natural gas consumption has fallen in most sectors, but the power sector has risen. When the emission reductions are 10, 20, and 30%, oil and gas consumption in the power sector has increased by 1.24, 2.21, and 2.66%, respectively. The electricity energy consumption in various sectors can also be seen in Figure 2. Overall, except for the electric power sector, the demand for electric energy in all sectors has declined. With the increase in emission reductions, the decline has increased. Taking the service industry as an example, when the emission reduction varies from 10 to 30%, the decline in electricity and energy consumption increases from 1.26 to 4.53%. When the emission reduction varies from 10 to 30% for the power sector, the power consumption will increase from 1.71 to 6.00%.

**Figure 3:**
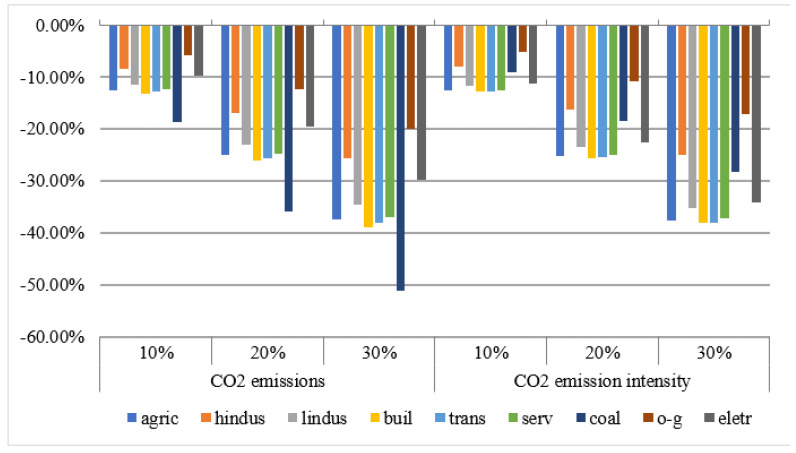
The sectors’ emissions and emissions intensity of carbon dioxide.

The changes in carbon dioxide emissions and emission intensity of various sectors are shown in Figure 3. The bars express the percentage change in sectors’ emissions and emissions intensity of carbon dioxide. It is obvious that the levy of a carbon tax reduces the carbon dioxide emissions of sectors. Among them, sectors with high demand for fossil energy, such as coal, have a significant reduction in carbon dioxide emissions, while oil and natural gas sectors have a relatively low reduction. The CO_2_ emission intensity of each sector is obtained by comparing the total CO_2_ emission of each sector with the sector’s nominal GDP. Due to the levy of carbon taxes, the total CO_2_ emissions of the sectors have decreased to varying degrees, but some sectors have increased for sector GDP. This leads to complex changes in the intensity of carbon dioxide emissions among sectors.

#### 5.1.3. Macroeconomic Variables Effects

The levy of carbon taxes lead to a decline in nominal GDP and real GDP, and with the continuous increase in emission reductions, the fall has gradually increased [41]. Table 3 shows the impact of carbon taxes on different macroeconomic variables. For households, the levy of a carbon tax leads to a rise in total income, and it increases with the emission reductions growth. The decline in households’ demand leads to a rise in households’ savings. With the increase of the carbon tax, social welfare has fallen more and more. The social welfare drops from −80.206 billion yuan to −295.336 billion yuan, when the emission reduction ranges from 10 to 30%. For enterprises, levying a carbon tax leads to a decline in income. Their savings have also fallen, and the decline is even greater with the increase in emission reductions. For the government, the main revenue comes from taxes. With the increase in carbon tax revenue, government revenue and savings have increased significantly. When carbon dioxide emissions are reduced by 10, 20, and 30%, government revenue will increase by 1.65, 3.43, and 5.4%, respectively. As emission reductions continue to increase, the reduction in carbon dioxide emission intensity gradually increases.

### 5.2. Carbon Tax Recycle Simulation Results

#### 5.2.1. The Impact on Institutions

The changes of various economic institutions in different simulation scenarios are depicted in Table 4. Regarding households’ income, under the four scenarios, households’ labor income remains unchanged, and the income of capital decreases. Households’ capital income fell by 0.43, 0.54, 0.42, and 0.28% under the baseline scenario and the three simulated scenarios. Therefore, as Table 4 shows, the total income of residents under the four scenarios declines, and compared with the baseline scenario, the decline in the simulated scenario is greater. Households’ demand level rose by 1.02% in scenario 1 and declined in other scenarios. Households’ savings level fell the most in scenario 1, which was 0.08%. In scenarios 2, 3, the decline was 0.06 and 0.04%, respectively. It can be seen from the baseline scenario that the levy of a carbon tax caused the social welfare of residents to drop to −1767.84. Compared with the baseline scenario, households’ social welfare in scenarios 1 imposes a carbon tax while reducing resident income tax and 3 imposes carbon tax while reducing corporate indirect tax under the carbon tax cycle improved to 3276.83 and −337.08.

For the enterprise, the income of the enterprise fell in all four scenarios. The decline rates of the baseline scenario and simulated scenarios 1, 2, and 3 are 0.43, 0.54, 0.42, and 0.28%, respectively. It is worth noting that under scenario 2, which is to impose a carbon tax while reducing corporate income tax, the income of enterprises has fallen, but due to the reduction of the corporate income tax rate, the level of corporate savings has been significantly increased. The corporate savings have increased significantly by 2.48%. In other scenarios, corporate savings have fallen. As for the government, the total revenue is fixed under these three simulation scenarios. Under scenarios 1 and 2, the government’s demand drops by 0.2 and 0.26%, respectively. However, in scenario 3, government demand rises by 0.29%.

In general, scenario 1 imposes a carbon tax while reducing residents’ income tax, which increases the level of residents’ demand and social welfare. Scenario 2 reduces the corporate income tax rate, increasing corporate savings but resulting in a more significant decline in residents’ consumption and income. Compared with the baseline scenario, the social welfare of residents has fallen even more. Scenario 3 reduces enterprises’ indirect tax rates while increasing government consumption. Compared with the baseline scenario where only a carbon tax is levied, residents’ social welfare has improved.

#### 5.2.2. The Impact on the Economy

The changes in GDP are shown in Table 5. Both nominal GDP and real GDP declined in the four scenarios. Compared with the baseline scenario, nominal GDP in scenario 3 has the largest decrease, which is 0.86%, scenarios 1 and 2 fall by 0.25 and 0.17%, respectively. Scenario 1 has a larger reduction of real GDP than the BaU, which is 0.28%. For total investment, scenario 2 increases by 1.21%. Scenarios 1 and 3 reduce by 0.48% and 0.24%, respectively.

#### 5.2.3. The Impact on Carbon Emissions

As shown in Table 5, under the premise of reducing carbon dioxide emissions by 20%, the baseline scenario’s carbon dioxide emission intensity has dropped by 19.84%. The changes in scenarios 1, 2, and 3 are almost the same as the BaU, with a decrease of 19.8, 19.86, and 19.30%, respectively, which can also achieve carbon emission reduction. Compared with the BaU, the carbon tax changes very little. The four scenarios’ carbon tax prices are 71.35, 71.88, 73.44, and 76.97 yuan/ton.

## 6. Discussion and Conclusions

### 6.1. Discussion

In this article, using the 2017 input–output table and other official data sources, we constructed a static CGE model with nine sectors to simulate the impact of China’s low-carbon economic policies on the social economy. The specific analysis includes the impact of carbon tax policies on energy consumption, carbon dioxide emissions, sectoral economic, and macroeconomic variables under different carbon dioxide emission reductions. Besides, we analyze the impact of carbon tax cycle mechanisms on macro-social economic variables under the principle of tax neutrality and test the “double dividend” theory.

When a carbon tax is imposed, the ad valorem tax rate levied on fossil energy gradually increases as the total emission reductions increase. Among them, the tax rate of coal is higher than that of oil and natural gas. The results are consistent with some previous studies [51,52]. Obviously, most of the contribution to emission reduction comes from coal, which is mainly due to the high carbon emission coefficient of coal and that coal occupies a dominant position in China’s energy consumption structure. With the gradual increase in CO_2_ emission reductions, energy consumption has gradually decreased, coal has the largest decline, while electricity has a smaller decline. According to Wang’s research findings [9], carbon dioxide emissions in China’s fossil energy consumption mainly come from coal, and the empirical results of this paper also confirm this conclusion. A carbon tax would have the biggest impact on coal than any other energy source. Therefore, companies will reduce the demand for coal to cut down production costs. As a secondary energy source, electricity consumes a large amount of fossil energy. Still, it is indirectly affected by collecting carbon taxes and does not directly emit CO_2_, so it has little effect on electricity consumption. From the impact of carbon taxes on different sectors, it is found that the demand for coal in sectors has dropped significantly, and the decline has increased with the gradual increase in emissions reductions. In terms of oil and gas consumption, the consumption in sectors other than the power sector has declined. The main reason is that the mutual substitution of input factors between sectors and the increase in other input factors’ prices for the power sector has led to a rise in oil and natural gas demand. Due to a carbon tax levy, sectors’ emissions have declined when total CO_2_ emission reductions gradually increase. Among them, sectors with high demand for fossil energy, such as coal, significantly reduce CO_2_ emissions.

Various scenario simulations have an impact on macroeconomic variables. In the case of only levying a carbon tax, with the gradual increase in emission reductions, the decline in macroeconomic variables such as GDP, real GDP, social welfare, and total corporate income gradually increase. The total income of residents and the government has gradually increased. Furthermore, to reduce the negative impact of the carbon tax, we simulated carbon tax recycling measures such as reducing indirect corporate tax and household income tax while collecting carbon tax. While levying carbon taxes, reducing residents’ income tax increases residents’ demand, thus increasing residents’ social welfare. Besides, imposing a carbon tax has improved the environment, thus realizing the “double dividend” of the carbon tax. While imposing carbon taxes, the corporate income tax rate is lowered. Because carbon taxes impact the production process, enterprises’ capital price and total income are still falling. However, due to the reduction of corporate income tax rates, corporate savings have increased significantly. The decline in residents’ capital income leads to a substantial decrease in residents’ income, savings, and consumption, which cause a decrease in residents’ social welfare and cannot realize the “double dividend” of the carbon tax. While levying carbon taxes, the corporate indirect tax rate is lowered. Since indirect taxes only occur in the distribution of domestic products, companies can pass the tax burden on to consumers, affecting domestic production product demand and demand prices. Therefore, corporate income and savings will decline; the decline in residents’ capital income will also decrease. However, due to the reduction of indirect taxes, the demand price of products has fallen. Therefore, the consumption demand of residents has risen. At the same time, compared with just levying a carbon tax, residents’ social welfare has increased.

### 6.2. Conclusions

The purpose of this study is to explore how the carbon tax policy that promotes the development of a low-carbon economy should be effectively implemented, so as to achieve green and sustainable development. Studies have found that carbon tax has a positive impact on reducing corporate carbon dioxide emissions, promoting the development of more energy-saving emission reduction technologies, and exploring more renewable resources, which is conducive to the realization of green and sustainable development goals. The research results also show that the levy of a carbon tax will adversely affect economic development, residents’ income, and social welfare. However, introducing a suitable carbon tax recycling mechanism when formulating carbon tax policies can reduce the impact of the carbon tax on related industries. The implementation of a carbon tax recycling system based on the principle of tax neutrality, on the one hand, is conducive to achieving carbon emission reduction targets. On the other hand, it can improve the level of social welfare of residents, so as to achieve the double dividend of carbon tax.

Implementing a reasonable carbon tax recycling system (such as reducing the personal income tax of residents when formulating carbon tax policy) can reduce carbon emissions, promote economic growth, and achieve the “double dividend” effect. Currently, China is implementing a structural tax reduction policy. Whether the carbon tax can be implemented well is a challenge and an opportunity to further improve China’s taxation system. The carbon tax system’s design must conform to the overall direction of “tax reduction and burden reduction”, by upholding the principle of tax neutrality, and gradually achieving the goal of green emission reduction.

Some limitations of this research will be addressed in future work. On the one hand, a dynamic CGE model will be established on the basis of the carbon tax static CGE model constructed in this article, so as to better reflect the long-term impact of the carbon tax policy. On the other hand, in future works, our research can be extended to analyze different market-based tools, such as tradable emission permits.

## Figures and Tables

**Table 1:** Scenarios setting.

Design Exploration	Scenarios	Description
Carbon tax policy	Business as Usual (BaU)	A carbon tax is levied on the intermediate input of energy in the production sector, and no carbon tax is levied on the final demand sector.
Carbon tax cycle	Scenario 1	Based on the BaU scenario, reduce the resident income tax rate and maintain government revenue neutrality.
	Scenario 2	Based on the BaU scenario, reduce the enterprise income tax rate and maintain government revenue neutrality.
	Scenario 3	Based on the BaU scenario, reduce the enterprise indirect tax rate and maintain government revenue neutrality ^1^.

^1^ Since the department’s indirect tax rate is not equal, an equal percentage reduces its indirect tax rate.

**Table 2:** The carbon tax and energy statistics.

Scenarios	Energy	10%	20%	30%
Carbon tax (yuan/ton)		30.50	71.35	128.20
Fossil energy tax rate	Coal	0.11	0.24	0.42
	O-G	0.03	0.06	0.11
Fossil energy reduction contribution	Coal	97.46%	96.87%	96.10%
	O-G	2.54%	3.13%	3.90%
Energy consumption change	Coal	−12.22%	−24.29%	−36.14%
	O-G	−1.26%	−3.10%	−5.78%
	Electricity	−0.44%	−0.92%	−1.46%

Note: O-G stands for oil and gas.

**Table 3:** Statistics of macroeconomic variables.

Scenarios	10%	20%	30%
Nominal GDP	−0.09%	−0.20%	−0.33%
Real GDP	−0.11%	−0.28%	−0.52%
Household income	0.03%	0.06%	0.08%
Household demand	−0.25%	−0.55%	−0.92%
Household saving	0.03%	0.06%	0.08%
Enterprise income	−0.20%	−0.43%	−0.70%
Enterprise saving	−0.20%	−0.42%	−0.70%
Government income	1.65%	3.43%	5.40%
Government saving	1.65%	3.43%	5.40%
CO_2_ emission intensity	−9.91%	−19.84%	−29.77%
Social welfare	−802.06	−1767.84	−2953.36

**Table 4:** Statistics of macroeconomic variables of institutions.

Heading	Heading	Scenarios			
		BaU	Scenario 1	Scenario 2	Scenario 3
Households	Social welfare	−1767.84	3276.83	−2203.59	−337.08
	Labor income	0.00%	0.00%	0.00%	0.00%
	Capital income	−0.43%	−0.54%	−0.42%	−0.28%
	Total income	0.06%	−0.08%	−0.06%	−0.04%
	Demand	−0.55%	1.02%	−0.69%	−0.11%
	Savings	0.06%	−0.08%	−0.06%	−0.04%
Enterprises	Total income	−0.43%	−0.54%	−0.42%	−0.28%
	Savings	−0.42%	−0.54%	2.48%	−0.28%
Government	Total income	3.43%	0.00%	0.00%	0.00%
	Demand	3.18%	−0.20%	−0.26%	0.29%

**Table 5:** Statistics of the economy and carbon emissions.

Heading	Scenarios			
	BaU	Scenario 1	Scenario 2	Scenario 3
Nominal GDP change	−0.20%	−0.25%	−0.17%	−0.86%
Real GDP change	−0.28%	−0.28%	−0.26%	−0.23%
Total investment change	−0.19%	−0.48%	1.21%	−0.24%
CO_2_ emission intensity change	−19.84%	−19.80%	−19.86%	−19.30%
Carbon tax (yuan/ton)	71.35	71.88	73.44	76.97

## Data Availability

Energy Information Administration (https://www.eia.gov/, accessed on 6 October 2021), China Financial Yearbook (https://www.epsnet.com.cn/, accessed on 6 October 2021), China Statistical Yeabook (http://www.stats.gov.cn/tjsj/ndsj/, accessed on 6 October 2021), and International Energy Statistics (https://www.eia.gov/, accessed on 6 October 2021).
